# Supplementary material for: An open-source framework for large-scale, flexible evaluation of biomedical text mining systems
Source: J Biomed Discov Collab. 2008 Jan 29;3:1. doi: 10.1186/1747-5333-3-1 (PMC2276192; doi:10.1186/1747-5333-3-1)
Supplement: Additional file 1 — Evaluation platform source code. The additional file contains the software evaluation framework discussed in this paper. The most current release can be downloaded from SourceForge.net [1]. See the accompanying README file for instructions on installation and use. Also included in the distribution is a collection of UIMA wrappers for some commonly used BioNLP tools and annotated corpora. [file 1747-5333-3-1-S1.zip › bionlp_uima_repository-1.0.1/docs/api/edu/uchsc/ccp/util/nlp/annotation/filter/OverlappingAnnotationResolver.html]

OverlappingAnnotationResolver (Center for Computational Pharmacology Util/UIMA API)


|  |  |  |
| --- | --- | --- |
| **Overview** \| **Package** \| **Class** \| **Tree** \| **Deprecated** \| **Index** \| **Help** | |  |
| PREV CLASS   NEXT CLASS | **FRAMES**    **NO FRAMES**     **All Classes** |  |
| SUMMARY: NESTED \| FIELD \| CONSTR \| METHOD | DETAIL: FIELD \| CONSTR \| METHOD |  |


---


## edu.uchsc.ccp.util.nlp.annotation.filter Class OverlappingAnnotationResolver

```
java.lang.Object
  edu.uchsc.ccp.util.nlp.annotation.filter.OverlappingAnnotationResolver
```

---

``` public class OverlappingAnnotationResolver extends Object ```

This class mirrors the functionality of the class by the same name in the edu.uchsc.ccp.uima package

**Author:**
:   williamb

---

**Constructor Summary**

`OverlappingAnnotationResolver(String annotationTypeToResolve)`


| **Method Summary** | |
| --- | --- |
| `protected  void` | `keepLongestAnnotation(List<TextAnnotation> annotations, Map<Integer,TextAnnotation> annotationIDMap, Map<Integer,Boolean> annotationIDRemovedMap, List<TextAnnotation> annotationsToRemove)`             keeps the longest annotation for a group of overlapping annotations |
| `List<TextAnnotation>` | `processAnnotations(List<TextAnnotation> annotations)` |

**Methods inherited from class java.lang.Object**

`clone, equals, finalize, getClass, hashCode, notify, notifyAll, toString, wait, wait, wait`

**Constructor Detail**

### OverlappingAnnotationResolver

```
public OverlappingAnnotationResolver(String annotationTypeToResolve)
```


**Method Detail**

### processAnnotations

```
public List<TextAnnotation> processAnnotations(List<TextAnnotation> annotations)
```

---


### keepLongestAnnotation

```
protected void keepLongestAnnotation(List<TextAnnotation> annotations,
                                     Map<Integer,TextAnnotation> annotationIDMap,
                                     Map<Integer,Boolean> annotationIDRemovedMap,
                                     List<TextAnnotation> annotationsToRemove)
```

:   keeps the longest annotation for a group of overlapping annotations

    :   **Parameters:**: `annotationIDMap` -: `annotationIDRemovedMap` -: `annotationsToRemove` -: `jcas` -


---


|  |  |  |
| --- | --- | --- |
| **Overview** \| **Package** \| **Class** \| **Tree** \| **Deprecated** \| **Index** \| **Help** | |  |
| PREV CLASS   NEXT CLASS | **FRAMES**    **NO FRAMES**     **All Classes** |  |
| SUMMARY: NESTED \| FIELD \| CONSTR \| METHOD | DETAIL: FIELD \| CONSTR \| METHOD |  |


---
